# Floral visitation of European honey bees and hoverflies in selected cultivated cucurbitaceous crops in Morogoro, Eastern-Central Tanzania

**DOI:** 10.1371/journal.pone.0322219

**Published:** 2025-05-12

**Authors:** Elvillah William Rweyemamu, Sija Kabota, George Muhamba Tryphone, Marc De Meyer, Maulid Walad Mwatawala

**Affiliations:** 1 Department of Crop Science and Horticulture, Sokoine University of Agriculture (SUA), Morogoro, Tanzania; 2 Research, Consultancy and Publication Unit, National Sugar Institute (NSI), Kidatu-Morogoro, Tanzania; 3 Royal Museum for Central Africa, Invertebrates Section and JEMU, Leuvensesteenweg 13, Tervuren, Belgium; Saint Xavier's College, INDIA

## Abstract

Cucurbit production in many parts of the world is constrained by the absence of effective pollinators. Due to the decline of European honey bees (*Apis mellifera*), main pollinators of cucurbits, there is a need to explore other cucurbits flowers visiting insects to supplement pollination services and for their conservation. Studies were carried out in the two agroecological zones of Morogoro Region, Eastern - Central Tanzania. We assessed visitation abundance, visitation frequency and visitation rate of four cucurbits flowers visiting insects under the family Syrphidae [*Eristalinus megacephalus, Mesembrius caffer, Paragus borbonicus* and *Toxomerus floralis*] and *A. mellifera* on cucumber (*Cucumis sativus*), watermelon (*Citrullus lanatus*) and squash (*Cucurbita moschata*). Field trials were conducted in a 5 × 3 × 2 × 2 factorial arrangement in a randomized complete block design for two seasons. Results showed significant effects of the interaction between agroecological zones × cucurbit species × season × flowers visiting insects on visitation abundance, visitation frequency and visitation rate (*p* < 0.0001) of flower visiting species. *Apis mellifera* was the dominant species in cucurbit flowers at both agroecological zones during the two growing seasons. Given its relatively higher visitation, *T. floralis* is a promising hoverfly species to be explored for its role in the pollination of *C. sativus* and *C. lanatus*. Agroecological zone, season and cucurbit species determined the visitation of European honey bees and hoverflies on cucurbits flowers. Cucurbit growers are suggested to employ management practices on farms that favour the abundance and foraging activities of these flower visiting insects for improved and sustainable cucurbit production.

## 1. Introduction

Insect pollination of flowering plants is significant in terrestrial environments, as it provides vital ecosystem services for human well-being such as crop production [1,2]. Insect pollinators (managed and wild), particularly species belonging to families Diptera, Hymenoptera, and Lepidoptera play a crucial role in over a third of global food production [2,3]. However, several studies have reported a decline in these pollinators due to abiotic and biotic factors [4–7]. Consequently, the production of insect-pollinated crops including cucurbits has been negatively impacted [8,9].

Cucurbit is a generic term for fruit vegetables that belong to the family Cucurbitaceae. They are among the most important plant families supplying humans with edible products rich in essential vitamins and minerals [10–12]. There is an increased demand for cucurbit products worldwide attributed to the consumers’ preferences motivated by health concerns [13–15]. However, the production of cucurbits is affected by several factors including insufficient pollination services [15–17].

Cucurbits are predominantly monoecious crops having male flowers with heavy and sticky pollen grains that cannot be carried easily by wind [8,18,19]. Thus, cucurbits are obligatorily dependent on insect pollinators, and in an open system, they show a significant pollination deficit [20]. Globally, European honey bees (*Apis mellifera* Linnaeus) are known to be the most efficient pollinating agents of cucurbits and are believed to contribute to nearly 80% of the total insect pollination community [21–23]. However, populations of *A. mellifera* are declining because of several factors including farming intensiﬁcation, application of agrochemical pollutants, urbanization, climate change and socio-cultural perceptions [6,24–31]. Thus, it is unlikely that the demand for pollination services by European honey bees in cucurbits can be sustained, and this may affect the transfer and deposit of pollen. Efforts to enhance the abundance of the European honey bee have so far yielded limited results, and hopes for sustainable pollination have been reduced [15,32]. Therefore, there is a need to investigate alternative flower visiting insects to supplement pollination services to cucurbits.

Cucurbits are visited by diverse insect pollinators whose abundance and distribution depend on various factors such as the efficiency of a visiting insect species [33], intra and inter-specific competition [34], flower density and morphology [35,36], quality of floral resources [37–41], abiotic environment [34], weather [42–48], altitude and landscape [49–55] and habitat heterogeneity [56–59]. Studies conducted in different parts of the world have reported hoverflies (Diptera: Syrphidae) as providers of ecosystem services such as pollination [60–62]. They rank second after bees by visiting 52% of global crop plants and constituting 19% of all insect pollinators [63–65]. Hoverflies exhibit a strong preference for yellow color, pollen and more rewarding flowers [66]. Their bodies are covered with hairs and bristles capable of carrying pollen over long distances since they don’t have a confined home range like the European honey bees [63]. Furthermore, most hoverflies are migratory and are capable of escaping non-conducive environments. Therefore, with the decline of the European honey bee populations, the arrivals and departures of hoverflies may play crucial roles in maintaining ecosystem services such as pollination and pest control [67–69]. However, the role of hoverfly species in cucurbit pollination is not well known. Sawe et al. [15] in northern Tanzania reported dominant pollinators in watermelon that include European honey bees (87.8%) followed by hoverflies (8.5%). A study by Kabota [62] reported hoverflies; [*Eristalinus megacephalus* Rossi, *Mesembrius caffer* (Loew), *Paragus borbonicus* Macquart and *Toxomerus floralis* (Fabricius)] as the most abundant species foraging on cucurbits. However, this study did not determine the preference of hoverflies among cucurbit species.

Therefore, this study aimed at investigating the floral visitation of four dominant hoverfly species; *M. caffer, E. megacephalus, P. borbonicus* and *T. floralis* along with *A. mellifera* on the selected cucurbits species: - cucumber (*Cucumis sativus* Linnaeus), watermelon [(*Citrullus lanatus* (Thunb.) Matsum. & Nakai)] and squash (*Cucurbita moschata* Duchesne). These cucurbit species were selected because they are commonly cultivated in the country as cash and food crops. We hypothesized that visitation abundance, visitation frequencies and visitation rate of the European honey bees and hoverflies are influenced by cucurbit species, season and agroecological zones. We expected the visitation abundance and visitation rate of flower visiting species to be lower on cucurbit species with low flower density and during dry season. The results of this study will help in providing understanding to farmers on other useful flower visiting insects for their conservation.

## 2. Materials and methods

### 2.1. Description of the study area

The study was conducted in two agroecological zones (i.e., Plateau and Mountainous zones) of Morogoro, Eastern-Central Tanzania located between latitudes 6° 49’ 49.3428’‘ S and longitudes 37° 40’ 14.1204’‘ E [70]. The Plateau zone has an elevation ranging from 200 m to 600 m.a.s.l, with annual rainfall ranging from 800 mm to 1000 mm and annual average temperature ranging from 19˚C to 31˚C. The Mountainous zone has elevation ranging from 800 m to 2000 m.a.s.l, with annual rainfall ranging from 1000 mm to 1200 mm and annual average temperature ranging from 10˚C to 25˚C. The region has a bimodal type of rainfall with short rains in October - December and long rains from March – May [71]. This study was conducted on the plots established under the Agroecological Methodology in Vegetable crops (AGROVEG) project with the permission approved by the project leaders.

### 2.2. Experimental design

Trials were conducted for two seasons from March to June 2021 (predominantly the rainy season) and September to November 2021 (predominantly the dry season). Four experimental plots of 45 m × 45 m each were established at each agroecological zone. The distance between plots was approximately 1 km. Each plot was divided into three subplots of 15 m × 45 m to accommodate the three cucurbit species. A Randomized Complete Block Design (RCBD) in a 5 × 3 × 2 × 2 factorial arrangement was used with four replications.

### 2.3. Crop establishment and observation of cucurbits flower visitors

Three cucurbit species were planted on 23^rd^ March 2021 and 2^nd^ September 2021 during the rainy and dry seasons, respectively. Agronomic practices such as weeding and irrigation were similar in all plots. Observations of European honey bees and hoverflies visitation in cucurbits commenced at 30–35 days (when 10% of the crops had flowered) and continued until the end of the blooming period. We established 8 spots, 4 m^2^ each at a spacing of 5 m on transects formed on each subplot per cucurbit species. We observed flower visiting insects once a week by walking on the transect at three time slots, from 0800 to 0900 hours, 1200–1300 hours and 1600–1700 hours. The time slots allowed observations of flower visiting insects with different diurnal activity patterns [72]. Each plot was allocated three observers, one on each cucurbit species. The observation was conducted simultaneously in all plots and for both agroecological zones. The sampling procedures were adopted from Zameer et al. [73] with a slight modification on the observation time.

On a 4 m^2^ spot, we counted the number of visits paid by each flower visiting species on a flower for one minute. We also counted flowers visited by each flower visiting species, including time (seconds) spent on a flower and time spent in flight between consecutive flowers. We determined the total number of opened flowers, average corolla height and corolla diameter per cucurbit species. We also recorded data on the weather (rainfall, relative humidity and temperature) during each day of sampling. A data logger (iButton, Maxim Integrated Products, Sunnyvale, CA, USA) was used to record relative humidity and temperature, rainfall was recorded using a rain gauge which was installed in one of the locations where experimental plots were established in the Mountainous zone. Weather data for the Plateau zone were obtained from the Tanzania Meteorological Authority (TMA).

Then, the following variables were processed:

Visitation abundance: - Total number of flower visiting insects per plot observed visiting each cucurbit species flowers within three minutes of observation regardless of its previous visits.

Visitation frequency**: -** A total number of visits by each flower visiting species per selected cucurbit flower. It was determined by closely observing a single flower for a maximum of one minute and recording the number of visits per flower visiting species. A total of 24 flowers per cucurbit species were observed per day.

Average number of visits on a single cucurbit flower per cucurbit species by individual pollinating species was determined following the method described by Zameer et al. [73]

$VF=\frac{TNV}{TFO}$ (i)

Where, VF is visitation frequency, TNV is total number of visits and TFO is total number of flowers observed.

Visitation rate**: -** Total number of flowers of each cucurbit species visited by each flower visiting insects within one minute. To establish visitation rate, an individual flower visiting insect was closely followed for a maximum of one minute from the moment it landed on the first flower. We then recorded for each flower visiting species, all flowers visited within a 4 m^2^ spot, time (seconds) spent on each visited flower and time spent in flight between consecutive flowers. Three visual observations were made for each flower visiting species per cucurbit species per day.

Average number of flowers visited per flower visiting species was determined following the formula described by Meerabai [74]:

$VR=\frac{TNFV}{TSF+TFBCF}\;$ (ii)

Where, VR is visitation rate, TNFV is the total number of flowers visited, TSF is the time spent on flowers and TFBCF is the time in flight between consecutive flowers.

### 2.4. Statistical analysis

We computed total number of visits of each flower-visiting species on the flowers of three cucurbits species for the whole study period. Then, we determined the effects of the agroecological zones, seasons and cucurbit species on visitation abundance, visitation frequency and visitation rate of European honey bees and hoverflies on the three cucurbit flowers. Analysis of variance (ANOVA) was performed using Generalized Linear Mixed Models (GLMMs) procedures at a significant level of 5%. GLMMs account for non-normal data that have both fixed and random effects and where data transformation can violate the assumptions of normality [75]. Normality test was performed using Shapiro-Wilk and the data did not conform to a normal distribution. Agroecological zones, seasons and cucurbit species were treated as fixed factors and sampling week as a random factor. Model selection was performed using Akaike’s information criterion. To validate the significance between the factors, the *post hoc* test was performed where the means were compared using Tukey’s HSD test at 5% level of confidence. All analyses were performed using R software version 4.1.0 [76].

## 3. Results

### 3.1. Visitation abundance

A total of 13171 visits of European honey bees and hoverflies were recorded on *C. sativus*, *C. moschata* and *C. lanatus* flowers for the whole study period in the Mountainous and Plateau zones of Morogoro in 8 fields (Table 1). *Apis mellifera* constituted 56.94% and hoverflies 43.06% number of visits of all flower visiting species on the three cucurbit species combined. By crop, 4992, 2776 and 5403 of all number of visits by flowers visiting species were observed on *C. sativus*, *C. moschata* and *C. lanatus* flowers, respectively. *Apis mellifera* recorded the highest number of visits on each cucurbit species; 3060, 1789 and 1789 followed by *T. floralis* 1186, 527 and 1724 for *C. sativus*, *C. moschata* and *C. lanatus*, respectively. The lowest number of visits on all the three cucurbit species were recorded for *M. caffer* 35, 38 and 58 *C. sativus*, *C. moschata* and *C. lanatus* flowers, respectively. For hoverflies, *T. floralis* was dominant (3437) followed by *P. borbonicus* (1099), *E. megacephalus* (1004) and lastly *M. caffer* (131).

**Table 1:** Visitation abundance of European honey bees and hoverflies in three cucurbit species.

Pollinators	Number of visits			Total	Proportion number of visits (%)
***C. sativus***	***C. moschata***	***C. lanatus***
*A. mellifera*	3060	1789	2651	**7500**	**56.94**
*E. megacephalus*	240	264	500	**1004**	**7.62**
*M. caffer*	35	38	58	**131**	**0.99**
*P. borbonicus*	471	158	470	**1099**	**8.34**
*T. floralis*	1186	527	1724	**3437**	**26.10**
**Total**	**4992**	**2776**	**5403**	**13171**	**100**
**Proportion number of visits (%)**	**37.90**	**21.08**	**41.02**	**100**	

### 3.2. Cucurbit flowers variables and weather parameters

The cucurbits flower variables under the study indicated in Table 2 shows average flower density found in 4 m^2^, average corolla height and corolla diameter of 10 flowers. During the May-June season, average temperature, relative humidity and rainfall recorded in the Mountainous zone ranged from 18˚C to 24˚C, 70% to 89% and 0 mm to 2.16 mm, respectively, in the Plateau zone ranged from 22˚C to 25˚C, 69% to 82% and 0 mm to 0.75 mm. During the October-November season, average temperature, relative humidity and rainfall recorded in the Mountainous zone ranged from 20˚C to 25˚C, 70% to 80% and 0 mm to 0.59 mm, respectively, in the Plateau zone ranged from 26˚C to 30˚C, 63% to 71% and 0 mm to 0.1 mm, respectively.

**Table 2:** Flowers variables of the three cucurbits under the study.

Flowers variables	Cucurbit species		
*C. lanatus*	*C. sativus*	*C. moschata*
Flower density/4 m^2^	97.29 ± 6.89	87.29 ± 9.21	21.00 ± 1.88
Corolla height (cm)	1.93 ± 0.07	2.21 ± 0.22	5.36 ± 0.13
Corolla diameter (cm)	2.17 ± 0.07	2.42 ± 0.25	7.07 ± 0.25

### 3.3. Temporal variation on the visitation abundance, frequency and rate

**Fig 1:**
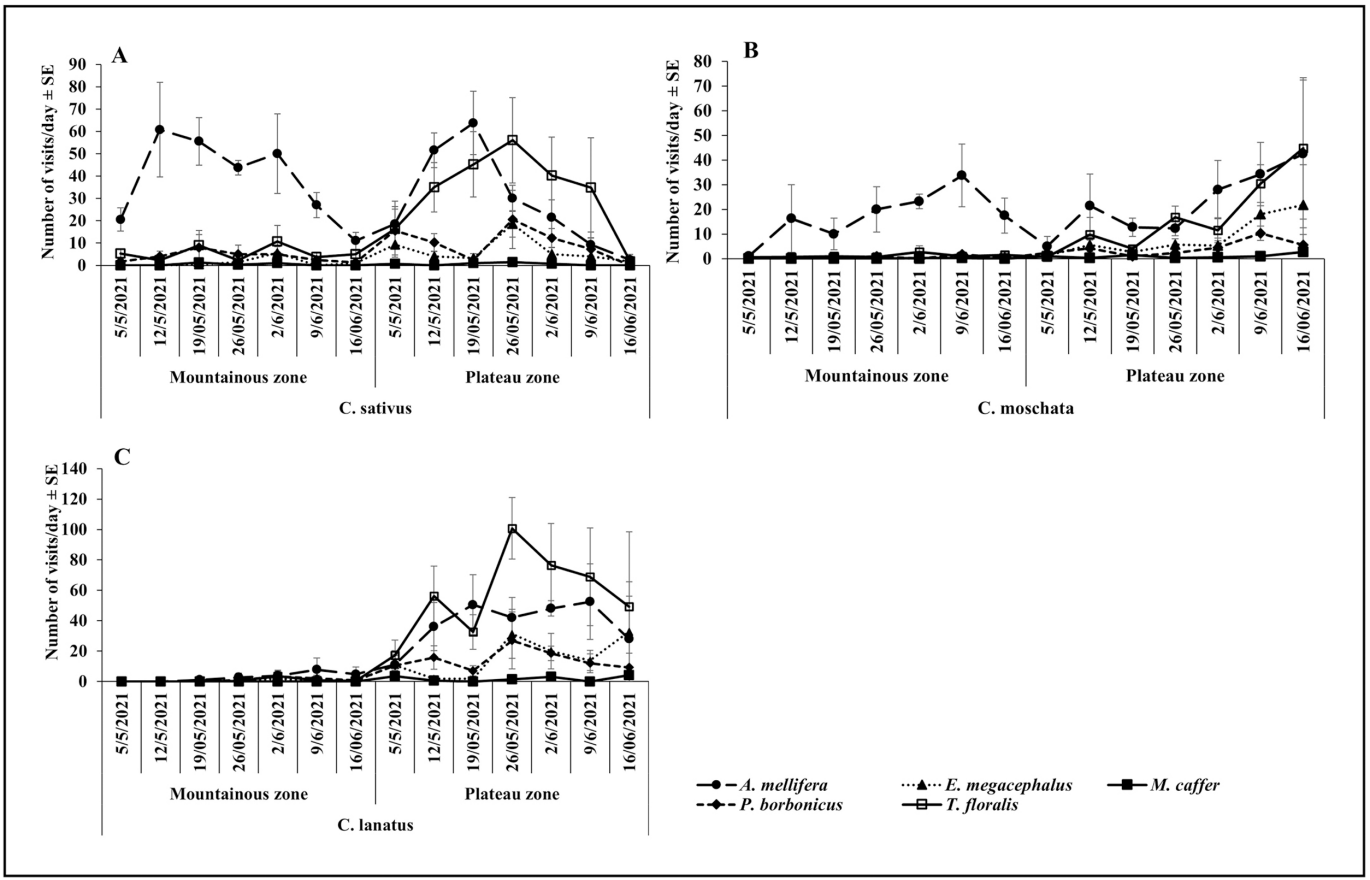
Temporal variation on the visitation abundance in the Mountainous and Plateau zone during the May-June.

**Fig 2:**
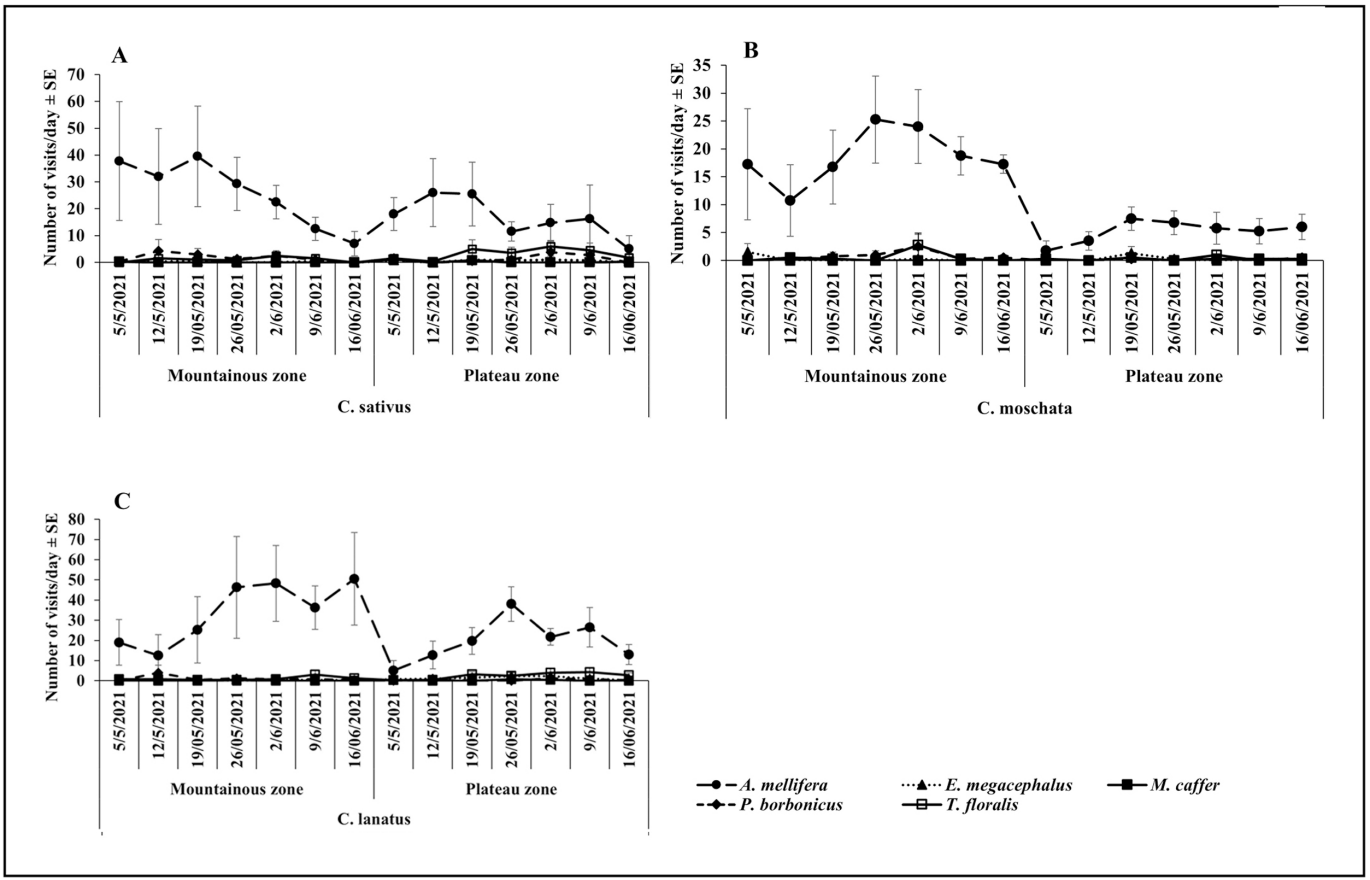
Temporal variation on the visitation abundance in the Mountainous and Plateau zone during the October-November.

The results showed that, during the May – June cropping season, *A. mellifera* was the most abundant visitor of all cucurbit species, while all other species were less prevalent (Fig 1A, B and C). Of all flowers visiting species, *M. caffer* was the least abundant visitor on all cucurbit species. There was a notable variation in the Plateau zone, where the visitation of *T. floralis* on *C. lanatus* increased considerably (100.75 ± 20.25) to exceed that of *A. mellifera* (Fig 1C). The trends recorded during the October-November season, showed dominance of *A. mellifera* in both zones and all cucurbit species. Visitation abundance of all other flowers visiting species remained very low (Fig 2A, B and C). Generally, abundance of prevalent species increased from the beginning and dropped towards the end cropping season (Figs 1A, B, C and 2A, B, C).

**Fig 3:**
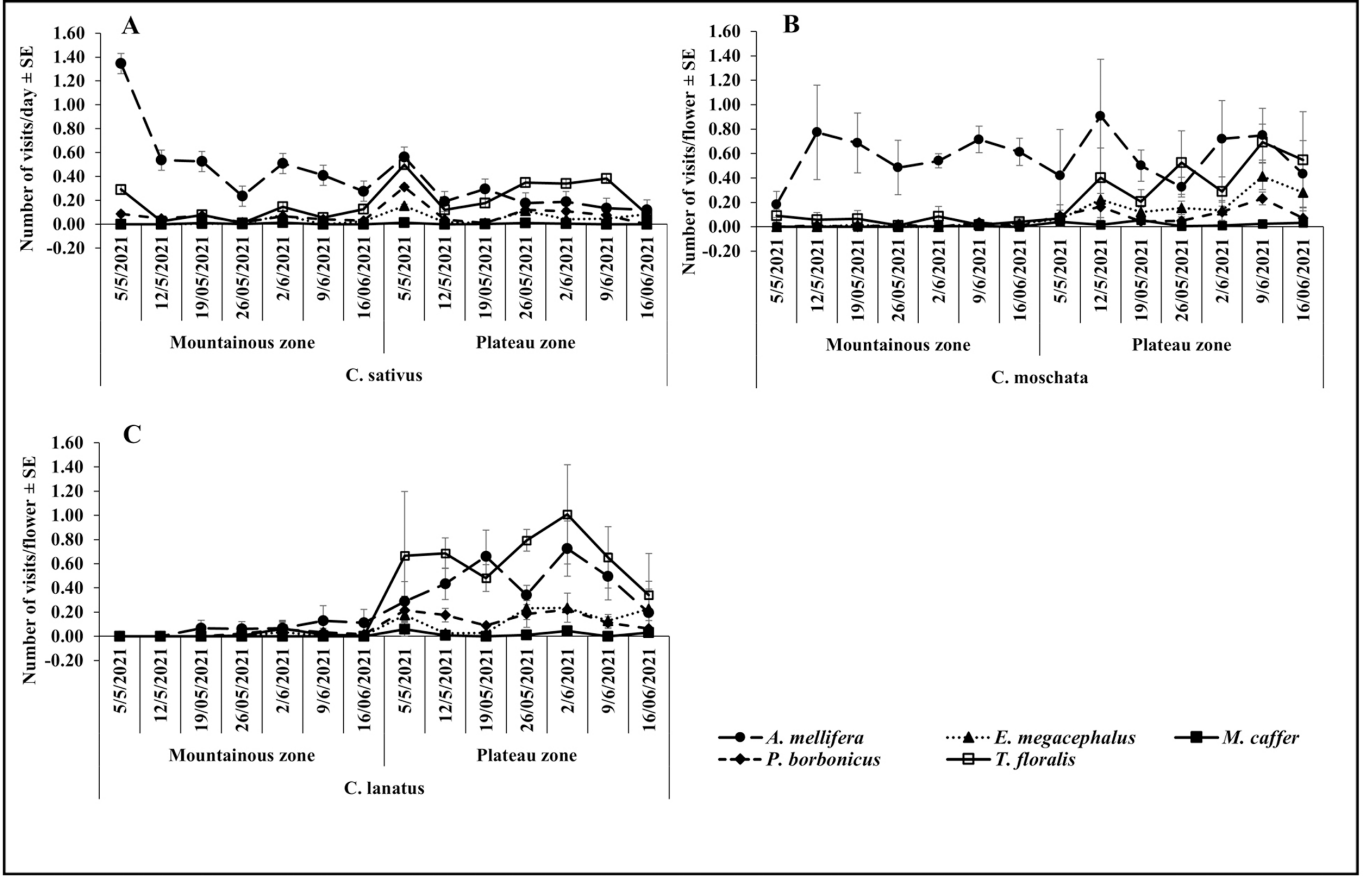
Temporal variation on the visitation frequency in the Plateau and Mountainous zones during the season of May-June.

**Fig 4:**
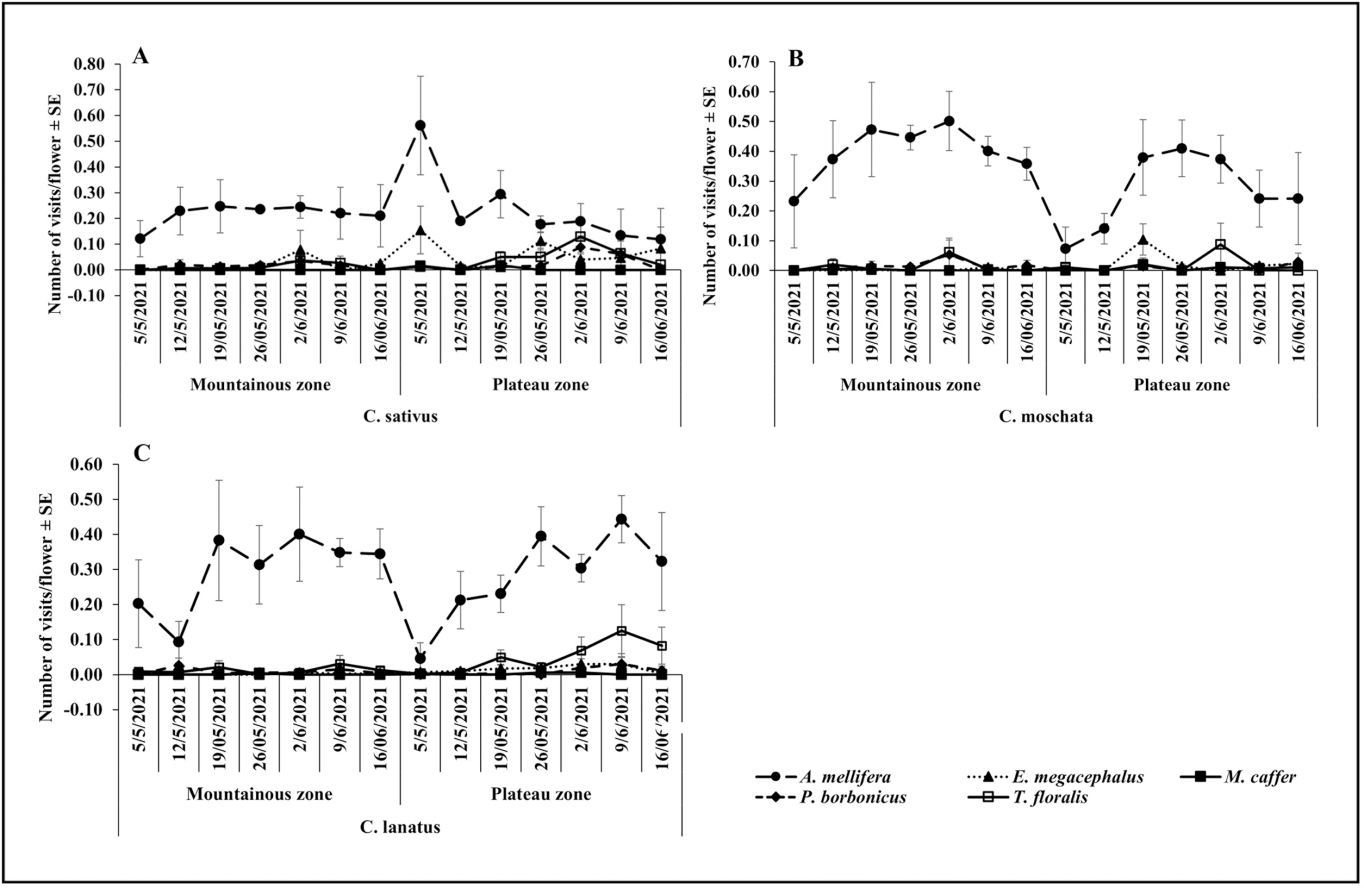
Temporal variation on the visitation frequency in the Plateau and Mountainous zones during the season of October-November.

**Fig 5:**
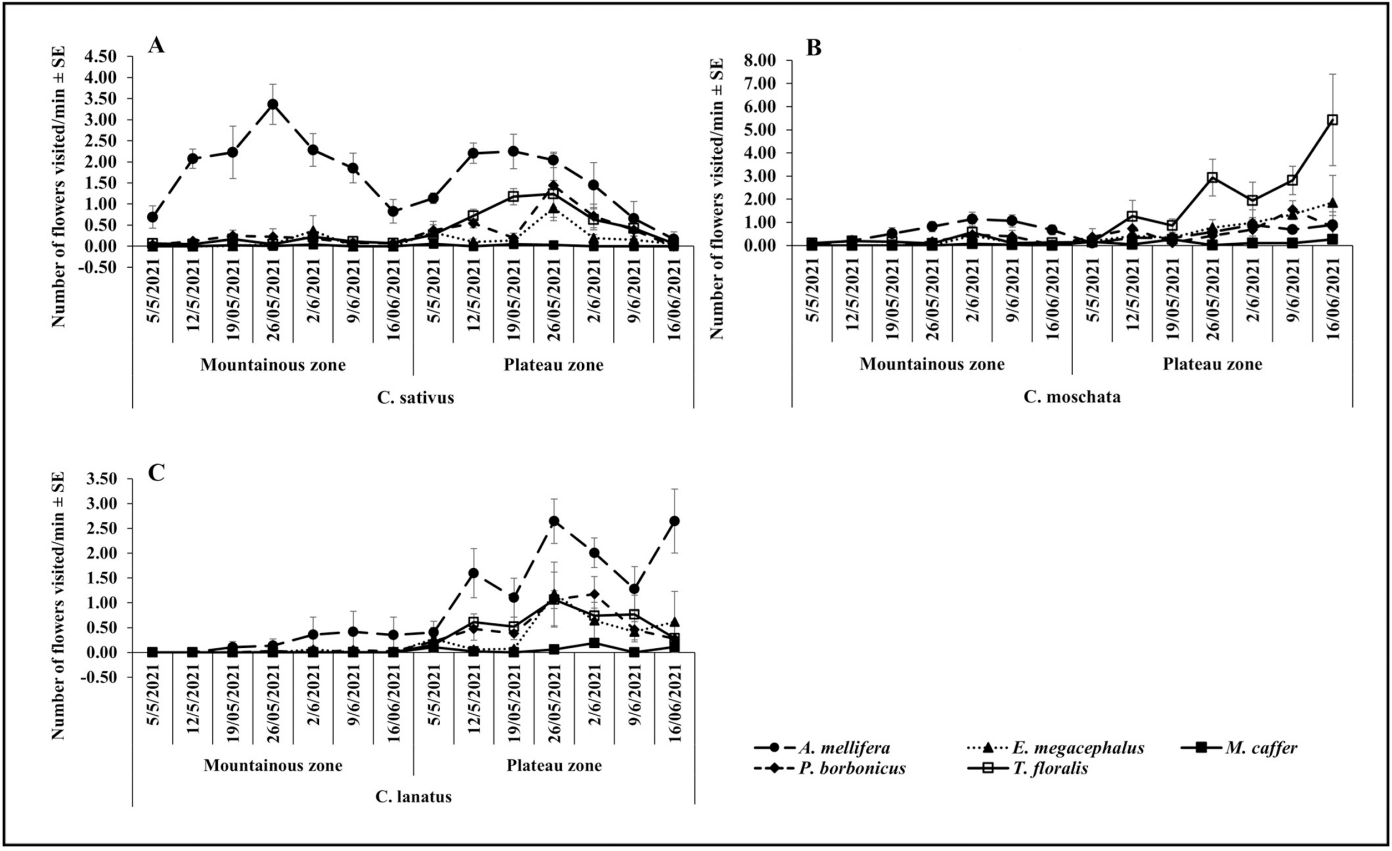
Temporal variation on the visitation rate in the Plateau and Mountainous zones during May-June season.

The visitation frequency of *A. mellifera* was generally higher than other flowers visiting species in both agroecological zones, all cucurbit species and during both seasons (Figs 3A, B, C and 5A, B, C). Visitation frequencies were relatively stable over time without any strong pattern during all cropping seasons and on all cucurbit species. Except for *A mellifera*, visitation frequencies of most species were very low and without any strong pattern during both seasons in the Mountainous zone. Frequency by *A. mellifera* increased from the beginning of the season, and dropped towards the end of the season in both agroecological zones, seasons and on all cucurbit species flowers (Figs 3A, B, C and 4A, B, C).

**Fig 6:**
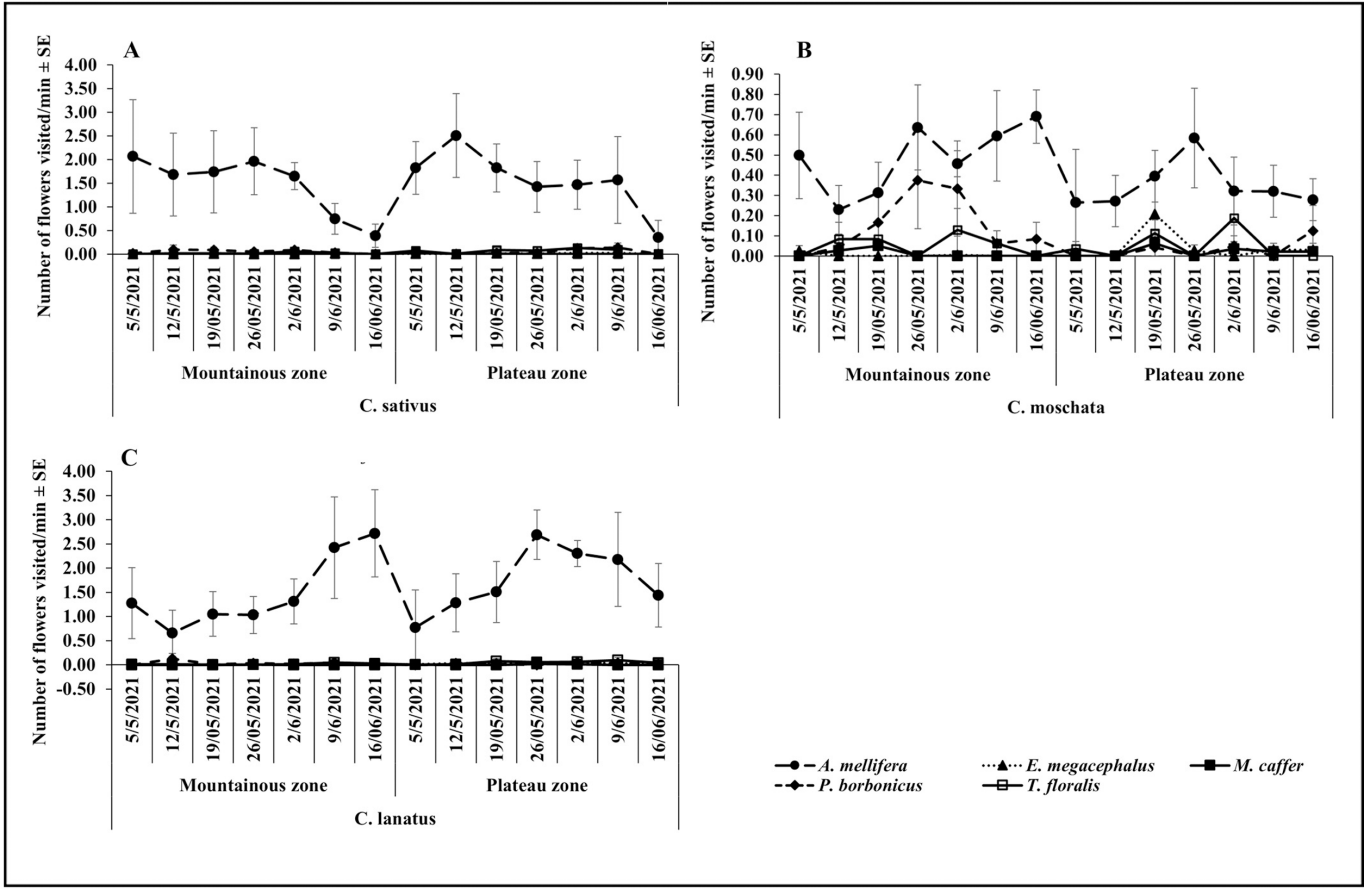
Temporal variation on the visitation rate in the Plateau and Mountainous zones during October-November season.

During the May-June season, *A. mellifera* visited the highest number of *C. sativus* flowers in both the plateau and mountainous zones (Fig 5A) and all cucurbit species during the October-November season (Fig 6A, B and C). The visitation rates of *E. megacephalus* and *P. borbonicus* on all cucurbits flowers were higher in the plateau zone during the May-June season (Fig 5A, B and C). The lowest number of flowers visited were recorded for *M. caffer* and the visitation patterns were fairly stable in both agroecological zones and seasons. In most cases, *A. mellifera* showed the highest visitation rate on cucurbits flowers with a noticeable pattern along the sampling weeks while other flowers visiting species’ visitation rates were extremely low, with no discernible pattern.

### 3.4. Effects of agroecological zone, season, cucurbit species and flowers visiting species on visitation abundance, frequency and rate

**Fig 7:**
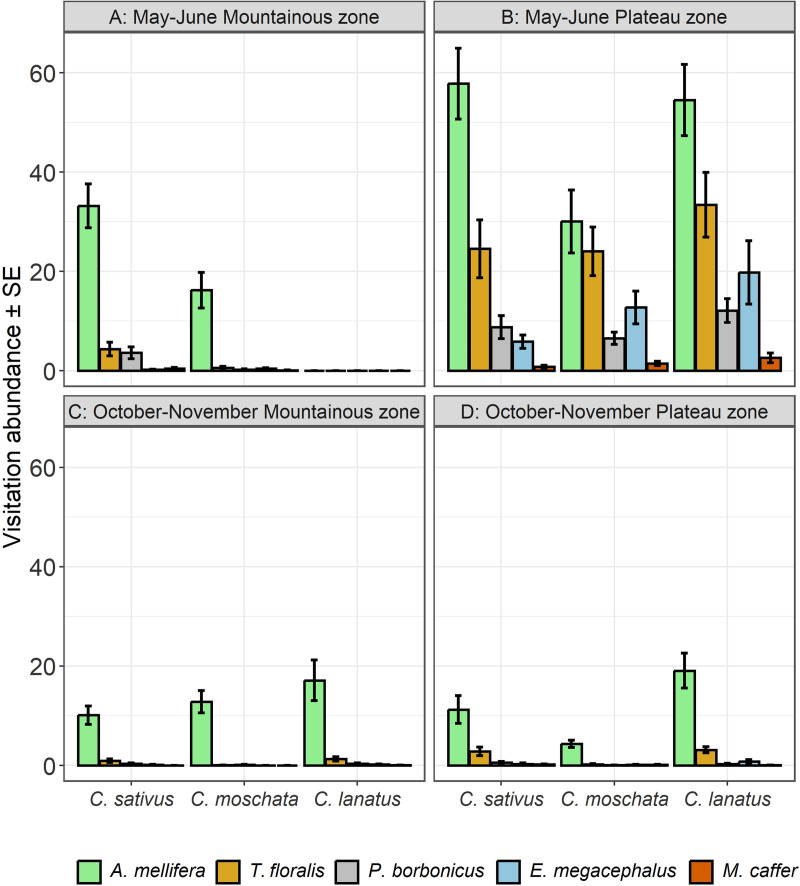
Visitation abundance on cucurbit flowers as affected by season, agroecological zones and cucurbit species.

There was a significant effect of agroecological zone × season × cucurbit species × flowers visiting species interaction on visitation abundance of European honey bees and hoverflies on cucurbit flowers (*p* < 0.0001). Also, all other factors and interactions were significant, except the season × cucurbit species interaction (Table 3). *Apis mellifera* recorded higher visitation on *C. sativus* during May – June season in the Plateau zone (Fig 7B) (*Post hoc* test, Tukey HSD). The lowest visitation abundance on all cucurbit species during both season and agroecological zones was recorded for *M. caffer* (Fig 7A, B, C and D).

**Table 3:** Effects of agroecological zones, seasons and cucurbit species on visitation abundance of European honey bees and hoverflies on cucurbit flowers.

Factors	Statistics		
**df**	**F value**	**P value**
Agroecological zone (Az)	1	51.19216	< 0.0001 ^***^
Season (Se)	1	86.7283	< 0.0001 ^***^
Cucurbit species (Cr)	2	17.0849	< 0.0001 ^***^
Flowers visiting species (Pol)	4	135.7771	< 0.0001 ^***^
Az × Se	1	108.5727	< 0.0001 ^***^
Az × Cr	2	17.25811	< 0.0001 ^***^
Az × Pol	4	20.33909	< 0.0001 ^***^
Se × Cr	2	1.658678	0.191 ns
Se × Pol	4	16.08716	< 0.0001 ^***^
Cr × Pol	8	4.429034	< 0.0001 ^***^
Az × Se × Cr	2	19.33815	< 0.0001 ^***^
Az × Se × Pol	4	15.24765	< 0.0001 ^***^
Az × Cr × Pol	8	3.404252	0.001 ^**^
Se × Cr × Pol	8	5.633144	< 0.0001 ^***^
Az × Se × Cr × Pol	8	4.475659	< 0.0001 ^***^

df: Degrees of freedom, significance codes: 0 ‘

***’ 0.001 ‘

**’ 0.01 ‘

*’ p ≥ 0.05 ^‘ns’^.

**Fig 8:**
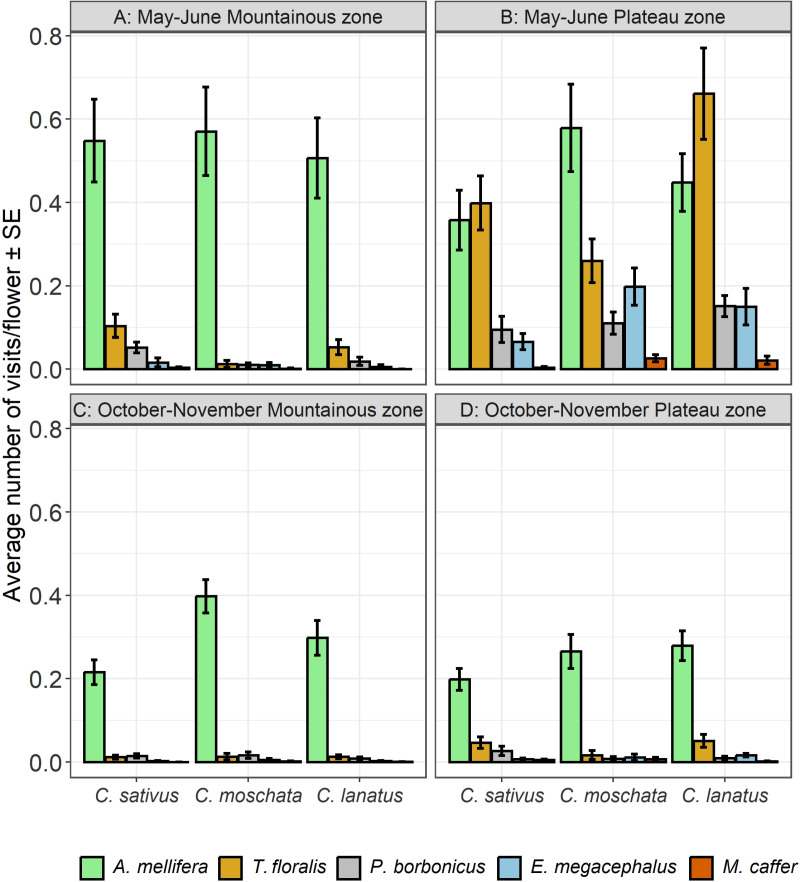
Visitation frequency on cucurbit flowers as affected by season, agroecological zones and cucurbit species.

Results showed significant effects of agroecological zone × season × cucurbit species × flowers visiting species interaction on visitation frequency of European honey bees and hoverflies on cucurbit flowers (*p* < 0.0001). The effects of all factors and interactions were also significant, except the season × cucurbit species interaction (Table 4). Further results (Fig 8A, B, C and D) showed significantly higher visitation frequency by *A. mellifera* on all cucurbit species than other flowers visiting species in both the agroecological zones and during both seasons (*Post hoc* test, Tukey HSD). A notable exception is the significantly higher visitation frequency by *T. floralis* on *C. lanatus* flowers, in the plateau zone during the May – June cropping season (Fig 8B). The frequencies of visits by all species were significantly higher in the Plateau zone than in the Mountainous zone during the May – June cropping season (Fig 6B). *Mesembrius caffer* showed the lowest visitation frequency on all cucurbit species, in both the agroecological zones and during both seasons (Fig 8A, B, C and D).

**Table 4:** Effects of agroecological zones, seasons and cucurbit species on visitation frequency European honey bees and hoverflies on cucurbit flowers.

Factors	Statistics		
**df**	**F value**	**P value**
Agroecological zone (Az)	1	45.917	< 0.0001 ^***^
Season (Se)	1	109.720	< 0.0001 ^***^
Cucurbit species (Cr)	2	5.603	0.004 ^**^
Flowers visiting species (Pol)	4	170.256	< 0.0001 ^***^
Az × Se	1	50.913	< 0.0001 ^***^
Az × Cr	2	18.277	< 0.0001 ^***^
Az × Pol	4	16.935	< 0.0001 ^***^
Se × Cr	2	1.508	0.222 ns
Se × Pol	4	16.035	< 0.0001 ^***^
Cr × Pol	8	7.394	< 0.0001 ^***^
Az × Se × Cr	2	18.537	< 0.0001 ^***^
Az × Se × Pol	4	10.204	< 0.0001 ^***^
Az × Cr × Pol	8	4.768	< 0.0001 ^***^
Se × Cr × Pol	8	4.938	< 0.0001 ^***^
Az × Se × Cr × Pol	8	4.337	< 0.0001 ^***^

df: Degrees of freedom, significance codes: 0 ‘

***’ 0.001 ‘

**’ 0.01 ‘

*’ p ≥ 0.05 ^‘ns’^.

**Fig 9:**
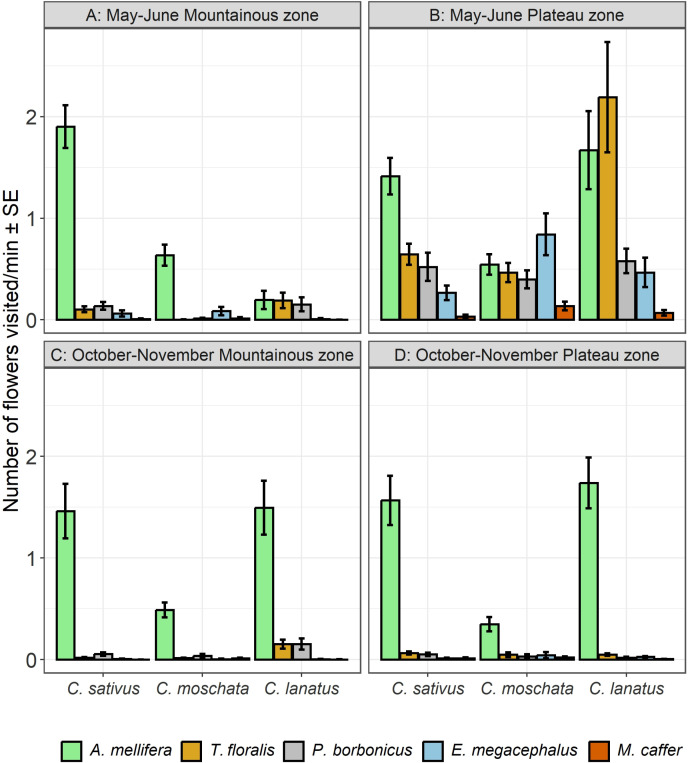
Visitation rate on cucurbit flowers as affected by season, agroecological zones and cucurbit species.

Our results also showed that all the factors and all the interactions had significant effects on the number of cucurbit flowers visited by flowers visiting species except cucurbit species (*p* = 0.080; Table 5). We therefore further examined the significant effects of agroecological zone × cucurbit species × flowers visiting species during the two seasons. Visitation rates were generally higher in the Plateau than the Mountainous zone during the May – June cropping season (Fig 9A and B) (*Post hoc* test, Tukey HSD). *Apis mellifera* had significantly higher visitation rates than the rest except *T. floralis* on *C. lanatus* in the Plateau zone (Fig 9A, B, C and D). Further results showed that *A. mellifera* was the most dominant species during the October – November cropping season, with a significantly higher number of *C. lanatus* and *C. sativus* flowers visited than *C. moschata* (Fig 9C and D).

**Table 5:** Effects of agroecological zones, seasons and cucurbit species on visitation rate European honey bees and hoverflies on cucurbit flowers.

Factors	Statistics		
**df**	**F value**	**P value**
Agroecological zone (Az)	1	57.415	< 0.0001 ^***^
Season (Se)	1	42.177	< 0.0001 ^***^
Cucurbit species (Cr)	2	2.523	0.080 ns
Flowers visiting species (Pol)	4	144.857	< 0.0001 ^***^
Az × Se	1	50.231	< 0.0001 ^***^
Az × Cr	2	6.351	0.002^**^
Az × Pol	4	6.211	< 0.0001 ^***^
Se × Cr	2	13.247	< 0.0001 ^***^
Se × Pol	4	13.757	< 0.0001 ^***^
Cr × Pol	8	23.678	< 0.0001 ^***^
Az × Se × Cr	2	8.044	< 0.0001 ^***^
Az × Se × Pol	4	6.317	< 0.0001 ^***^
Az × Cr × Pol	8	6.825	< 0.0001 ^***^
Se × Cr × Pol	8	4.603	< 0.0001 ^***^
Az × Se × Cr × Pol	8	4.644	< 0.0001 ^***^

df: Degrees of freedom, significance codes: 0 ‘

***’ 0.001 ‘

**’ 0.01 ‘

*’ p ≥ 0.05 ^‘ns’^

## 4. Discussion

Our results revealed the overall dominance of European honey bees over hoverflies on visitation abundance. The general number of visits of European honey bees was higher than hoverflies on all three cucurbit species. *Mesembrius caffer* recorded the lowest visitation abundance on all cucurbits species. We attributed this dominance to the population structure of *A. mellifera*. Honey bees maintain high populations relative to other species in many flower-visiting insect communities [77]. According to Wignall et al. [78], European honey bees can even create artificially high densities and exert competitive pressure on floral resources to other flower-visiting insects. In some cases, they forage over extensive ranges [79] and deplete floral resources [80]. These explain why European honey bees were more abundant visitors than hoverflies.

Temporal visitation abundance, visitation frequency and visitation rate of European honey bees and hoverflies on cucurbits flowers did not vary considerably along the sampling weeks, except *A. mellifera* and in a few cases *T. floralis*. The visitation of *A. mellifera* increased at the beginning and dropped at end of the season. This is because, most of cucurbits start flowering at 30–45 days after planting. As the plants grow, the number of flowers also increases and the abundance of flower visiting insects increases. This situation last for 8–10 weeks when fruits start to set [11]. The results of this study are in consistent with the findings of some of the research which reported that higher visitation abundance of flower visiting species is influenced by the presence of high flower density at the foraging site [73,81] and the foraging distance is reduced when there is high floral resources [82].

We found significant effects of interactions of agroecological zone, season, cucurbit species and flower visiting species on visitation abundance, visitation frequency and visitation rate of European honey bees and hoverflies. Our study allows the examination of three different but related concepts of visitation. Our discussion is therefore based on attributes of a visited flower versus a flower visiting species as affected by season and agroecological zone. *A. mellifera* was significantly dominant and frequent flowers visitor than other species in most cases. Previous studies reported that European honey bees are good foragers and primary pollinators of cucurbits [83–85]. An efficient pollinator must visit several flowers of the same species in succession and move frequently from one flower to another. Hoverflies do not consistently work the same on flowers as bees [33]. European honey bees have higher requirements for pollen and nectars because they live in colonies with large populations. Workers carry food for the entire brood, drones and queen. In contrast, flies require food for individual uses. Also, honey bees remain close to their colonies while flies are not limited in their foraging range [78].

Flowers of *C. lanatus* hosted more visitors than other cucurbits. A notable difference was low visitation rate of *A. mellifera* in *C. moschata* and the high visitation rate of *T. floralis* in *C. lanatus*. Flower morphology, density, and size determine the choice of flower visiting insects. Most of cucurbits have cup- or bell-shaped flowers with different corolla size and height [86]. Compared to flower density among three cucurbit species, *C. lanatus* had a higher flower density followed by *C. sativus* and lastly *C. moschata*. Previous studies reported the presence of high flower density increases visitation of flowers visiting species on a crop since it reduces competition between and within flowers visiting species and provides hoverflies (larvae and adults) a good habitat for breeding sites [38,87–90]. European honey bees have relatively long proboscis compared to non-specialists flies. The latter prefer to forage on open bowl flowers with short corollas for easy accessibility of floral rewards [55,91] and for laying eggs since emerging larvae are believed to feed on pollen [53]. Klecka et al. [92] classified *T. floralis* under small syrphids whose foraging behavior is influenced by morphological features of flowers such as corolla length, corolla diameter and floral shape. However, there were few variations. For example, the lower visitation rate of *A. mellifera* on *C. moschata* flowers can be explained by the study of Jachuła et al. [93] who reported that flowers with long corolla produce more nectars which makes European honey bees spend longer time foraging on a single flower.

We further examine the agroecological zone × season effects, as it qualifies the flower visiting species and cucurbit species effects. Our studies were conducted in two agroecological zones differentiated by altitude. The Mountainous (high altitude) zone and the Plateau (a low altitude) zone. European honey bees had higher visitation on all cucurbits in both agroecological zones and during all seasons. Habitat heterogeneity and environmental variables affect the distribution and abundance of insect pollinators [57–59,87]. According to Štípková et al. [94], Goodwin et al. [95] and Tarakini et al. [96], season and elevation influence the distribution and population of pollinators. Bees prefer to forage on the lower (warmer) altitudes while flies prefer the higher (colder) altitudes, especially in temperate and subtropical regions [97]. Previous studies showed hoverfly species abundance is highest during colder months [97–99], while European honey bees increase their activity during warmer periods of the year [100]. Thus, hoverflies are especially abundant during the colder time of the year when other native floral visitors are less active [97]. Our results showed dominance over seasons and agroecological zones. The reason could be altitudinal variation (500 m.a.s.l.) and seasonal weather changes were within the ranges of favorable conditions for the European honey bees.

## 5. Conclusions

In conclusion, our findings revealed that *A. Mellifera* was the most abundant and frequent visitors in all cases than hoverflies. However, higher visitation of honey bee may not necessarily have a high impact on pollination since they carry more pollen to feed the brood, queen and drones, whereas hoverflies can be effective pollinators since they only require floral resources for individual energy. This study further confirms results by Sawe et al. [15] that honey bees are dominant pollinators of in watermelon in northern Tanzania, followed by hoverflies. We further confirm results by Kabota [62] that among hoverflies, *E. megacephalus*, *M. caffer*, *P. borbonicus* and *T. floralis* as the most abundant species foraging on cucurbits. Hoverflies can play a complimentary pollination role to bees. However, further studies are needed to confirm the role in pollination and check the pollination efficiency of these flowers visiting species on cucurbits. Conservation of alternative (potential) pollinators is necessary because European honey bees remain important pollinators but their impact is under threat because of their declining populations as reported by other studies.
